# Multiple proliferation signaling pathways are modulated by octacalcium phosphate in osteoblasts

**DOI:** 10.7150/ijms.77017

**Published:** 2022-09-25

**Authors:** Yoona Jung, Jooseong Kim, Sukyoung Kim, Shin hye Chung, Jinhong Wie

**Affiliations:** 1 Department of Physiology, Konkuk University School of Medicine, Chungju, Republic of Korea.; 2 Dental biomaterials science, School of dentistry and dental research institute, Seoul National University, 101 Daehak-ro, Jongno-gu, Seoul, Republic of Korea.; 3 HudensBio Co., Ltd, 318 Cheomdanyeonsin-ro, Buk-gu, Gwangju 61088, Republic of Korea.; 4 Department of Biomedical Engineering, Yeungnam University, Daegu 42415, Republic of Korea.

**Keywords:** Octacalcium phosphate, Cell signaling pathway, Cell proliferation, ALP activity

## Abstract

Octacalcium phosphate (OCP), a type of bioactive ceramics, may be associated with dentine, tooth apatite, and especially bone generation, and promotes wound healing after fracture. Recently, commercial bone grafting products containing a large amount of OCP material have been released because OCP can be synthesized in large quantities. It is reported to increase cell proliferation, but the interaction between OCP and cell signaling pathways is still unclear. In this study, first, we demonstrated OCP mediated cell signaling pathways with only purified OCP materials. OCP regulated P38, JNK (c-Jun N-terminal kinase), Src, and AKT (protein kinase B) signaling pathways. OCP crystals appeared in the characteristic ribbon shape but varied by several tens of micrometers in size. The X-ray diffraction pattern was the same as previously reported. We studied two concentrations of OCP (10 mg/ml and 20 mg/ml) to understand whether the effect of OCP on cell signaling pathways is dose dependent. We confirmed that OCP treatment affected cell proliferation and alkaline phosphatase and disrupted Src phosphorylation but did not change the total protein level. P38 phosphorylation was activated with OCP treatment and inhibited by SB203580, but P38 total protein level did not change. OCP inhibited JNK phosphorylation signaling, whereas PD98509 inhibited JNK phosphorylation with or without OCP. Interestingly, the AKT total level decreased after OCP treatment, but AKT phosphorylation increased considerably. Our results demonstrate that OCP materials modulate cell signaling pathways and increase cell proliferation.

## Introduction

Bone regeneration takes a long time (6-12 weeks). During this period, the complicated physiological process of bone formation and fracture healing occurs. During bone remodeling in guided bone regeneration, the surface of the graft material is degraded, replaced, and finally resorbed. The resorption rate of implanted material is an important factor to consider because the remaining graft material may negatively affect the mechanical properties of newly-formed bone.

Currently, researchers are developing materials to shorten the bone remodeling time. One of these materials is calcium phosphate. Albee suggested bone defect repair using calcium phosphate and attempted to inject it into the gap between bones, which helped bone regeneration and union 4. Octacalcium phosphate (OCP; Ca_8_H_2_(PO_4_)_6_·5H_2_O) promotes both new bone production by osteoblasts and biodegradation by osteoclast-like cells 1-3. OCP granules activate osteoblasts on the bone surface and are involved in biological resorption by osteoclast-like cells 4, 5. OCP is widely used as a resorbable calcium phosphate ceramic bone graft material. It has osteoconductivity and bioactive properties 1-3. In addition, OCP has been proposed as a precursor of bone and tooth apatite and directly converts to hydroxyapatite (HA) 2, 6-12. The OCP crystal structure comprises alternative apatitic layers separated by hydrated layers. The apatitic layers have atomic arrangements of calcium and orthophosphate ions similar to those of HA, and the hydrated layers also similar to those of dicalcium phosphate dihydrate (DCPD) 8-11. Previous studies reported that OCP and HA differ structurally, leading to the higher osteoconductivity of OCP compared with that of HA, which affects osteoblast, osteoclast, and osteocyte functions 2, 3, 6.

However, despite OCP having osteoconductive and biocompatible properties 3, 13, only very small amounts of high-purity OCP can be obtained after synthesis due to thermodynamic instability and phase change. This leads to difficulties with mass production, and so OCP has not been widely used. As technology for synthesizing high-purity OCP continues to be developed, various attempts have been made to use OCP as bone graft material and scaffolds. Recently, OCP has been practically applied as a replacement material for human oral bone defects 14. However, further studies on how OCP affects intracellular signaling pathways are needed because only a few signaling pathways have been revealed to be associated with OCP, such as the P38/mitogen-activated protein kinase (MAPK) signaling pathway in stromal ST-2 cells 13 and JNK signaling pathway in bovine chondrocytes 15. Furthermore, even though OCP is mainly used for bone generation, studies on bone cells are scarce.

In this study, we investigated OCP-mediated intracellular signaling pathways were examined for the first time. Previous researchers used OCP mixtures, which were reported to increase cellular proliferation and wound healing. We hypothesized that pure OCP was also associated with cellular signaling transduction. Previous research found that OCP promoted cell proliferation and alkaline phosphatase (ALP) activity in MG63 cells 13. Based on these results, OCP may influence the signaling pathway. We studied whether OCP regulates four pathways: P38, JNK, Src, and AKT signaling pathways. We discovered that OCP increased P38 activity but disrupted JNK and Src signaling pathways. OCP also increased AKT phosphorylation but decreased AKT protein levels. These results may explain how OCP increases cell proliferation and wound healing through cellular signaling pathways.

## Materials and Methods

### Cell culture

MG63 cells (from Seoul National University) were maintained at 5% CO_2_ and 37 °C. Cells were cultured in DMEM (Gibco, Waltham, MA, USA), supplied with 1× penicillin-streptomycin and 10% fetal bovine serum (FBS) (Gibco).

### Alkaline phosphatase activity

The ALP activity was assessed using an alkaline phosphatase assay kit (Colorimetric) (Abcam, Cambridge, UK, ab83369) according to manufacturer's protocol. The detection kit was used to assess ALP levels in MG63 cells seeded on OCP and MG63 cells without OCP to evaluate the propensity of treated samples to stimulate biomineralization activity in cells. The method is based on ALP-dephosphorylating p-nitrophenyl phosphate (used as a phosphatase substrate) and changing its color (yellow, λmax = 405 nm).

The absorbance of the tested solutions was measured at 405 nm using a SpectraMax® ABS PLUS Absorbance Microplate Reader (Molecular Devices, LLC, Sunnyvale, CA, USA). After 5, 7, 11, and 13 days of incubation, ALP activity was used to assess the osteogenic activity of MG63. All measurements were performed in triplicate.

### Methylthiazolyldiphenyl-tetrazolium bromide (MTT) assay

Cell viability and proliferation was studied by MTT assay after 5, 7, 11, and 13 days of cell seeding on OCP and control cells (cells without OCP). MG63 cells were seeded into 96-well plates at a density of 3 × 10^5^ and incubated at 37 ºC and 5% CO_2_. The absorbance of each well was then measured using an ELISA plate reader (Sunrise™ Tecan, Austria) at 595 nm. All measurements were replicated three times to ensure reproducibility.

### Octacalcium phosphate crystal preparation

OCP powder was prepared using a heterogeneous synthesis method with DCPD (CaHPO_4_·2H_2_O, Junsei Co. Ltd., Japan). After adding 5 g of DCPD as a starting material to 1,000 ml of distilled water, it was heated while slowly stirring at 200 rpm using a magnetic stirrer until it reached 80 °C. Then, the pH was adjusted to 5. OCP started to precipitate after a few minutes, and the reaction was complete in approximately 10 min. The resulting precipitate was filtered using Whatman filter paper no. 2, washed with ethanol, and dried in an oven at 80 °C for 24 h.

### OCP plate preparation

The OCP powder was treated in each well (10 mg/ml or 20 mg/ml) of a 6-well cell culture plate with media for 24-48 h at 37 ºC and 5% CO_2_. After incubation, the cells were seeded in DMEM with 10% FBS and 1× penicillin-streptomycin at a density of 3 × 10^5^ in 6-well plates.

### The p38, and JNK inhibitors

SB203580 (30 μM, Sigma-Aldrich®, St. Louis, MI, USA) and PD98509 (25 μM, Selleckchem, TX, USA) were used in this study.

### Western Blotting

The cell lysate was centrifuged at 15,000 × g for 30 min at 4 ºC. The protein concentration was measured in the supernatant after centrifuging. Proteins were eluted for 10 min at 95 ºC with 3× lithium dodecyl sulfate buffer (diluted with RIPA buffer) supplemented with β-mercaptoethanol. Proteins were separated by SDS-PAGE on 10% gels using the mini-Protean Tetra handcast system® (Bio-Rad, California, USA) and transferred onto PVDF membranes by electroblotting. The membranes were blocked for 1 h in 5% nonfat dry milk in phosphate buffered saline-Tween20 (PBS-T). After washing with PBS-T twice, the membranes were incubated with the primary antibody (1:1000) overnight at 4 °C. After five washes with PBS-T, the membranes were incubated with anti-rabbit IgG, HRP-linked antibody (1:4000) or anti-mouse IgG, HRP-linked antibody (1:4000). Protein bands were detected using SuperSignal™ West Femto Maximum Sensitivity Substrate (34095). Primary antibodies used were: anti-P38 (1:1000), anti-phospho-P38 (1:1000), anti-SAPK/JNK (1:1000), anti-phospho-SAPK/JNK (1:1000), anti-phospho-Src (1:1000), anti-Src (1:1000), anti-AKT (1:1000), anti-phospho-AKT(Ser473) (1:1000), and anti-β-actin (1:1000). All antibodies were purchased from Cell Signaling Technology (Danvers, MA, USA).

### Statistical analysis

Image J was used for all protein level analyses (US National Institutes of Health). Results were compared using unpaired two-tailed t-tests between two groups. A *p*-value < 0.05 was considered to indicate a significant difference. All statistical analyses represented one standard error of the mean (mean ± s.e.m).

## Results

### Characteristics of the Octacalcium phosphate crystals

**Figure 1:**
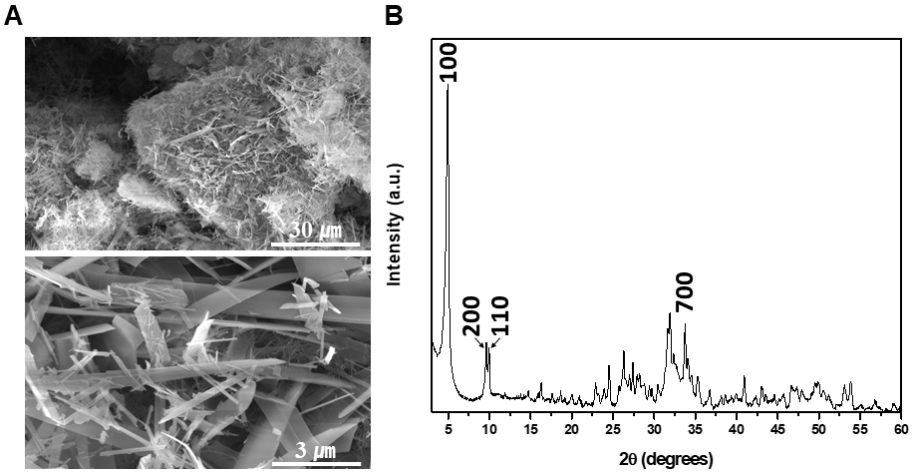
Scanning electron microscopy (SEM) and X-ray diffraction (XRD) patterns of synthesized octacalcium phosphate (OCP) powder: **(A)** SEM images (X1,000, X10,000) of OCP powder. All OCP powders appeared in a ribbon shape, but the size of the powders differed in the range of several tens of micrometers: **(B)** XRD pattern of the OCP powder. The peaks indicated which phase the sample is in, and this is an index that can be compared with the previously reported OCP. The presence of a high-purity OCP phase was confirmed.

The chemical composition of OCP was found to have a Ca/P molar ratio of 1.36 through FE-scanning electron microscopy (SEM)-energy dispersive spectroscopy measurement. SEM and X-ray diffraction (XRD) patterns of synthesized OCP are shown. The characteristic OCP shape is a ribbon, whose size differs by several tens of micrometers. In particular, the characteristic peak of (100) reflection was prominent (Fig. 1). In the XRD image, specific peaks of OCP were detected, indicating that the OCP was of high purity.

### Octacalcium phosphate crystals increased cell proliferation and stimulated biomineralization activity in MG63 cells

**Figure 2:**
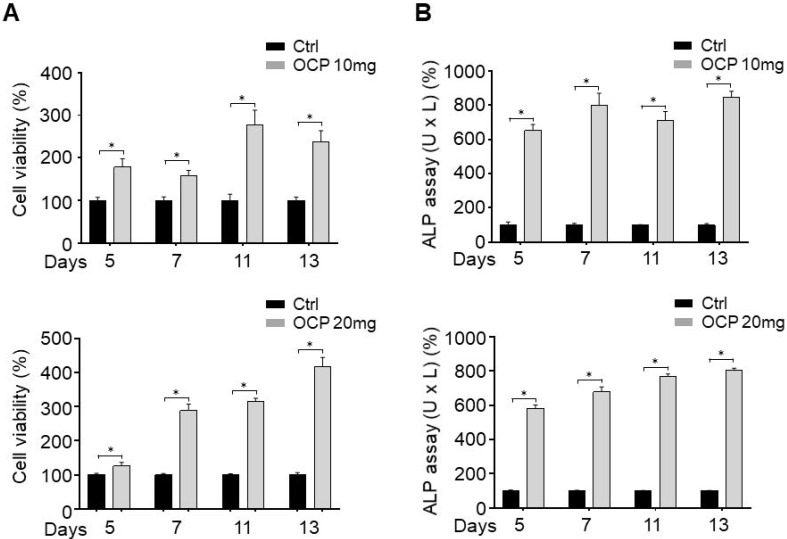
MTT assay **(A)** and Alkaline phosphatase (ALP) activity **(B)** of MG63 cells was measured with or without octacalcium phosphate (OCP) (10 mg/ml and 20 mg/ml). The graph represents results obtained at 5, 7, 11, and 13 days of incubation with or without OCP treatment. Data are means ± s.e.m. *p <0.05, n = 3, unpaired two-tailed t-test.

First, we investigated whether OCP increases cell proliferation using an MTT assay. The MTT assay results after 5, 7, 11, and 13 days showed that the cell proliferation of OCP-treated cells (10 mg/ml and 20 mg/ml) was upregulated; the difference between the cell proliferation of the control and experimental group was significant (*p* < 0.05) (Fig. 2A).

We carried out an ALP assay to confirm that OCP promotes the biomineralization activity of MG63 cells. The ALP activity of MG63 cells cultured with OCP (10 mg/ml, and 20 mg/ml) significantly increased (*p* < 0.05) (Fig. 2B). These results suggest that OCP can increase the activity of osteoblasts by upregulating factors that increase cell viability, cell proliferation, and biomineralization activity in MG63 cells.

### OCP processing facilitates the P38 signaling pathway

**Figure 3:**
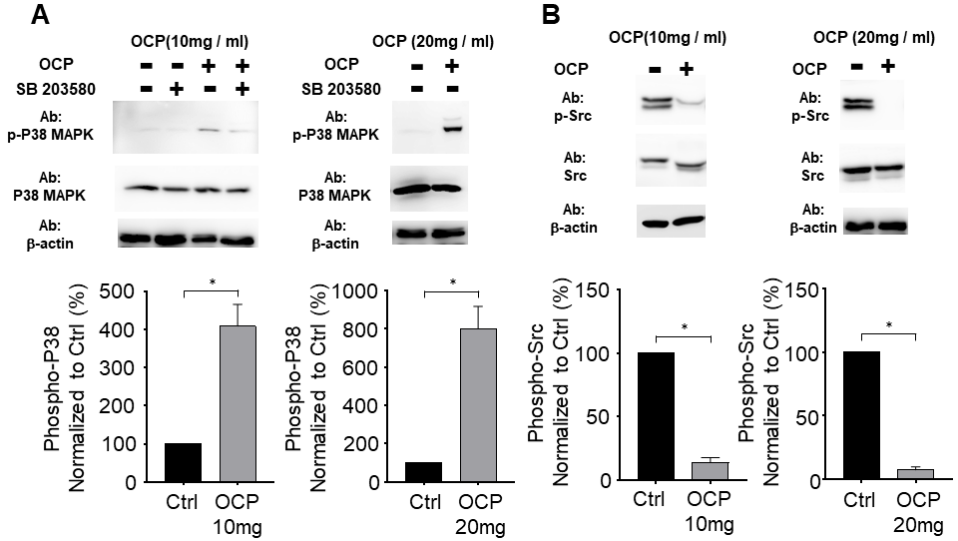
The octacalcium phosphate (OCP) treatment regulated P38 phosphorylation **(A)** and Src signaling pathway **(B)**. SB203580 (30 µM) used for P38 inhibitor. The bottom graph shows results normalized to the control. Data are means ± s.e.m. *p <0.05, unpaired two-tailed t-test.

We tested whether OCP activated the P38 pathway by growing MG63 cells in the presence of 10 mg/ml or 20 mg/ml of OCP. P38/MAPK pathway inhibitor (SB203580) treatment was used for the confirmation. There was no difference in P38 total protein levels between cells with or without OCP treatment. However, phosphorylation of P38 was significantly higher in the groups exposed to 10 mg/ml or 20 mg/ml OCP than in the control group with no OCP exposure (*p* < 0.05). Furthermore, OCP-induced phosphorylation of P38 was inhibited by SB203580 (30 µmol/L) (Fig. 3A). These results indicate that OCP activates the P38 signaling pathway in MG63 cells.

### Octacalcium phosphate crystals inhibit Src signaling in MG63 cells

We measured Src protein expression to check if OCP impacts the Src signaling pathway in MG63 cells. The total amount of Src protein did not differ with or without OCP treatment. However, when cells were cultured with OCP, phosphorylated Src had significantly lower protein expression than that of control group (*p* < 0.05) (Fig. 3B). These findings show that OCP inhibits the Src signaling pathway.

### Octacalcium phosphate attenuates the JNK signaling pathway by inactivating phosphorylated JNK

**Figure 4:**
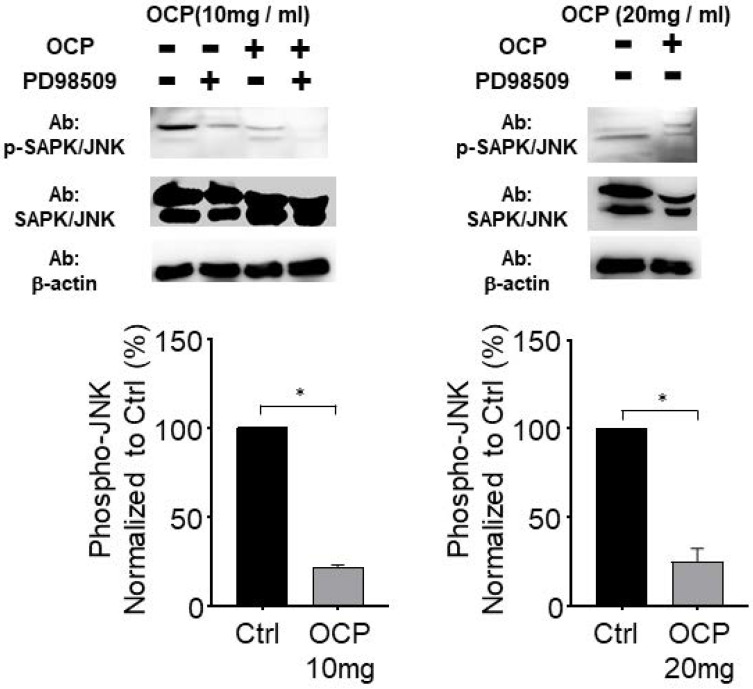
The JNK signaling pathway effect on Octacalcium phosphate (OCP) treatment. Lysates from MG63 cells were either treated with OCP (10 mg/ml or 20 mg/ml) or without OCP treatment. PD98509 (25 µM) used for JNK inhibitor. The bottom graph shows results normalized to the control. Data are means ± s.e.m. *p <0.05, unpaired two-tailed t-test.

We quantified the activated form of JNK, phosphorylated JNK (p-JNK), in MG63 cells to study the mechanistic pathway of OCP. p-JNK expression was significantly lower with the 10 mg/ml and 20 mg/ml OCP doses than in the control group (*p* < 0.05). Furthermore, OCP-induced-phosphorylation of p-JNK was completely inhibited by PD98509, the JNK inhibitor, (25 µmol/L) (Fig. 4). These results indicate that the JNK signaling pathway was disrupted by OCP by inhibiting JNK phosphorylation, which suggests that OCP may affect cell proliferation by inactivating JNK signaling, a key signaling system for apoptosis.

### AKT phosphorylation was promoted by octacalcium phosphate

**Figure 5:**
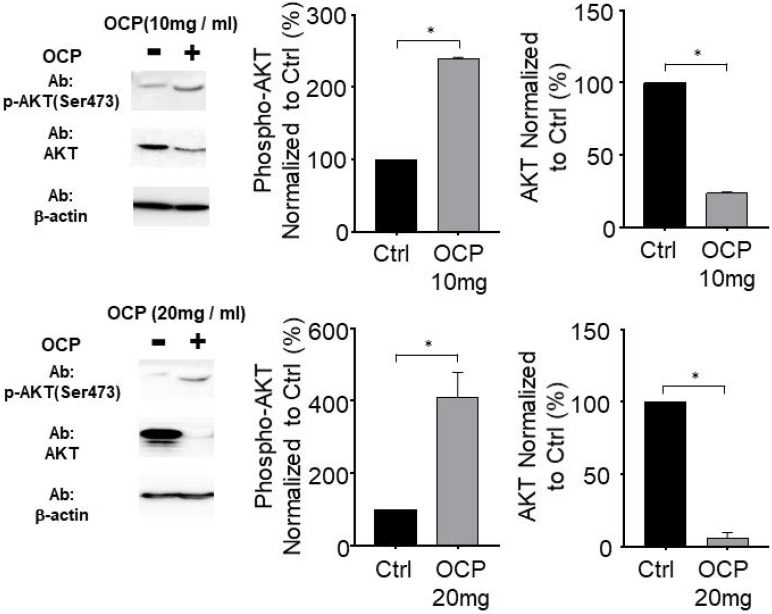
AKT (protein kinase B) signaling pathway regulated by octacalcium phosphate (OCP) (10 mg/ml and 20 mg/ml). The bottom graph shows results normalized to the control. Data are means ± s.e.m. *p <0.05, unpaired two-tailed t-test.

We incubated MG63 cells with OCP (10 mg/ml or 20 mg/ml) to determine whether OCP affects AKT signaling pathways. With OCP treatment, AKT phosphorylation significantly increased in MG63 cells (*p* < 0.05). However, the total AKT protein decreased significantly after OCP treatment compared to that in the control (*p* < 0.05) (Fig. 5). These results indicate that OCP increases the efficiency of AKT activation through activation of AKT S473 phosphorylation in the AKT signaling system. Therefore, it is shown that OCP plays a positive role in cell survival and proliferation through activation of the AKT S473 phosphorylation pathway in MG63 cells.

## Discussion

In this study, we revealed that OCP controlled the cell function mechanism. Previous research on OCP focused on wound healing or material composition. However, to the best of our knowledge, there has yet to be a sufficient explanation reported for why OCP increases cell signaling pathways. In the present study, the OCP treatment increased cell proliferation and viability. OCP increased the cell proliferation by more than 2-fold (10 mg/ml), and 3-fold (20 mg/ml) after 11 days (Fig. 2A). This result indicated that OCP accelerates the proliferation signaling pathway. We measured ALP activity and confirmed that its considerable increase was due to OCP (Fig. 2B). Cell proliferation is a complicated process, so we checked the four types of pathways involved. First, MAPKs regulate some key transcription factors that influence cell differentiation and functions in osteoblasts and osteoclasts 16. P38 is a member of MAPK, which is a family of enzymes that process extracellular stimuli. They contain a Ser/Thr kinase domain that is activated by phosphorylation by other Ser/Thr kinases. Thus, P38/MAPK phosphorylation impacts cell proliferation and differentiation. Here, OCP treatment increased P38/MAPK phosphorylation in cells compared to those without it. SB203580 is an P38/MAPK inhibitor, and it decreased P38/MAPK phosphorylation but did not affect the base protein level (Fig. 3A). Nishikawa et al. reported that OCP accelerated P38 phosphorylation in other cells 13. As OCP is a type of phosphocalcium ceramics formed of calcium and phosphorus at a ratio of 1.36, its hydrolysis to calcium-deficient HA started when it was implanted. When OCP characteristics changed, it reported to absorb calcium and export a little amount of phosphate, which promoted osteoblast cell proliferation 17.

Second, programmed cell death (JNK) is important in numerous intracellular biological processes, including immune response, tissue homeostasis, and normal cell turnover 18. OCP treatment decreased JNK phosphorylation in MG63 cells. JNK phosphorylation also decreased in MG63 cells with or without OCP after adding the PD98509 inhibitor (Fig. 4). Third, Src family tyrosine kinases regulate diverse cellular signaling pathways, including those involved in cell proliferation, morphology, survival, and cytoskeletal reorganization 20. Src activity is important for maintaining bone homeostasis. This signaling also regulates metastasis, invasion, and growth 21. Src phosphorylation controls various cellular processes including cell differentiation, survival signaling, migration, and proliferation. Src phosphorylation is widely understood as an important regulatory pathway, but the mechanisms involved are not fully understood. In the present study, Src phosphorylation signaling was interrupted by OCP in MG63 cells (Fig. 3B). Previously, Src has been reported to inhibit osteoblast differentiation 22, which explains why OCP increased cell proliferation.

AKT plays a key role in multiple connected signaling mechanisms involved in cell growth, metabolism, and apoptosis 23. Disruptions to AKT signaling are associated with neurological disorders, cancer, diabetes, and cardiovascular disease. AKT phosphorylation helps to increase cell proliferation 24. In the present study, AKT expression levels in OCP-treated cells decreased compared to those in cells without OCP treatment. However, interestingly, OCP rapidly promoted AKT phosphorylation. Hence, OCP treatment increased cell proliferation through increasing AKT phosphorylation (Fig. 5). However, how OCP decreased total AKT protein and increased AKT phosphorylation requires further investigations. Salinomycin and proteasome were reported to reduce total AKT protein and increase AKT phosphorylation 25, but this is not an effect of bioceramic materials. One possibility is that when the OCP is hydrolyzed, it becomes calcium-deficient HA. Furthermore, calcium and phosphate influence the AKT signaling pathway, but the specific mechanism remains unclear, so further studies are needed in future.

## Figures and Tables

**Scheme 1:**
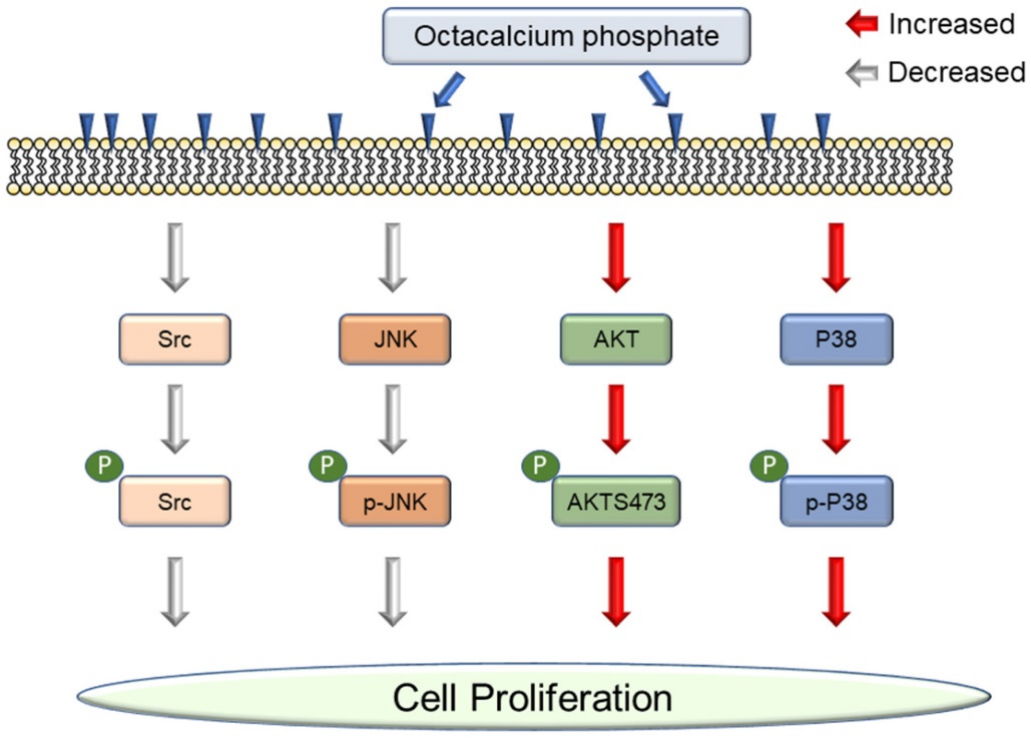
OCP modulated cellular signaling pathways.
